# Association mapping and marker-assisted selection of the lettuce dieback resistance gene *Tvr1*

**DOI:** 10.1186/1471-2229-9-135

**Published:** 2009-11-23

**Authors:** Ivan Simko, Dov A Pechenick, Leah K McHale, María José Truco, Oswaldo E Ochoa, Richard W Michelmore, Brian E Scheffler

**Affiliations:** 1 United States Department of Agriculture-Agricultural Research Service, Crop Improvement and Protection Research Unit, 1636 East Alisal Street, Salinas, CA 93905, USA; 2 The Genome Center and Department of Plant Sciences, University of California, 451 Health Sciences Drive, Davis, CA 95616, USA; 3 Rijk Zwaan BV, PO Box 40, 2678 ZG De Lier, the Netherlands; 4 Department of Horticulture and Crop Science, Ohio State University, Columbus, OH 43210, USA; 5 United States Department of Agriculture-Agricultural Research Service, Genomics and Bioinformatics Research Unit, 141 Experiment Station Road, Stoneville, MS 38776, USA

## Abstract

**Background:**

Lettuce (*Lactuca saliva *L.) is susceptible to dieback, a soilborne disease caused by two viruses from the family *Tombusviridae*. Susceptibility to dieback is widespread in romaine and leaf-type lettuce, while modern iceberg cultivars are resistant to this disease. Resistance in iceberg cultivars is conferred by *Tvr1 *- a single, dominant gene that provides durable resistance. This study describes fine mapping of the resistance gene, analysis of nucleotide polymorphism and linkage disequilibrium in the *Tvr1 *region, and development of molecular markers for marker-assisted selection.

**Results:**

A combination of classical linkage mapping and association mapping allowed us to pinpoint the location of the *Tvr1 *resistance gene on chromosomal linkage group 2. Nine molecular markers, based on expressed sequence tags (EST), were closely linked to *Tvr1 *in the mapping population, developed from crosses between resistant (Salinas and Salinas 88) and susceptible (Valmaine) cultivars. Sequencing of these markers from a set of 68 cultivars revealed a relatively high level of nucleotide polymorphism (*θ *= 6.7 × 10^-3^) and extensive linkage disequilibrium (*r*^2 ^= 0.124 at 8 cM) in this region. However, the extent of linkage disequilibrium was affected by population structure and the values were substantially larger when the analysis was performed only for romaine (*r*^2 ^= 0.247) and crisphead (*r*^2 ^= 0.345) accessions. The association mapping approach revealed that one of the nine markers (Cntg10192) in the *Tvr1 *region matched exactly with resistant and susceptible phenotypes when tested on a set of 200 *L. sativa *accessions from all horticultural types of lettuce. The marker-trait association was also confirmed on two accessions of *Lactuca serriola *- a wild relative of cultivated lettuce. The combination of three single-nucleotide polymorphisms (SNPs) at the Cntg10192 marker identified four haplotypes. Three of the haplotypes were associated with resistance and one of them was always associated with susceptibility to the disease.

**Conclusion:**

We have successfully applied high-resolution DNA melting (HRM) analysis to distinguish all four haplotypes of the Cntg10192 marker in a single analysis. Marker-assisted selection for dieback resistance with HRM is now an integral part of our breeding program that is focused on the development of improved lettuce cultivars.

## Background

**Figure 1:**
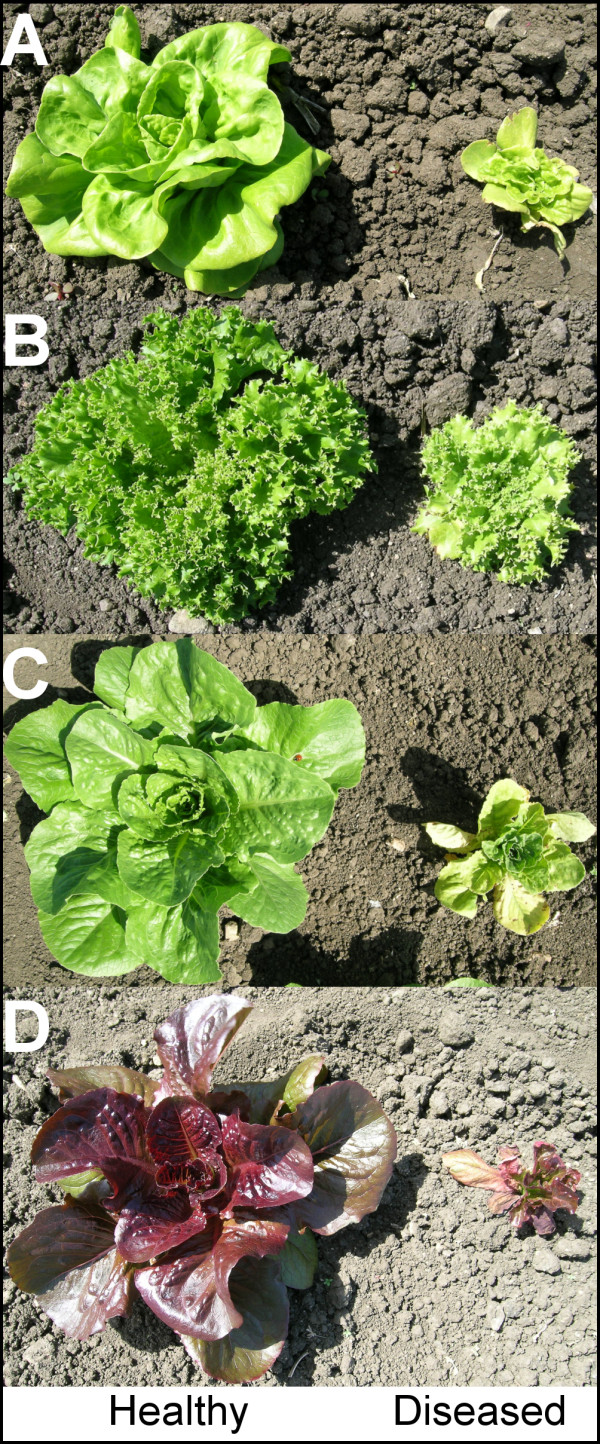
**Dieback symptoms on different types of lettuce: A - stem type, B - leaf type, C - green romaine, and D - red romaine**. Plants on the left are healthy, while plants on the right show typical symptoms of dieback, such as stunted growth, yellowing of older leaves, and gradual dying. Photographs were taken eight weeks after planting.

Lettuce dieback disease is widespread in commercially grown romaine and leaf-type lettuces [1]. The disease is caused by two closely related soilborne viruses from the family *Tombusviridae *-- Tomato bushy stunt virus (TBSV) and Lettuce necrotic stunt virus (LNSV) [2]. Symptoms of lettuce dieback include mottling and necrosis of older leaves, stunting, and plant death (Figure 1). The characteristic symptoms usually appear after the plant has reached 6 to 8 weeks of age and render the plant unmarketable [1]. TBSV and LNSV are extremely persistent viruses and they are likely to survive in soil and water for long periods of time [3]. The virus has no known vector and it seems to move through infested soil and water [4]. While fungal vectors are not necessary for transmission, studies have yet to be conducted to determine if such vectors can facilitate or increase rates of virus transmission to lettuce. Previous studies have provided no evidence that either chemical treatment or rotation with non-host crops can effectively reduce, remove, or destroy the virus in infested soil [5]. Since there are no known methods to prevent the disease in a lettuce crop grown in an infested field, genetic resistance remains the only option for disease control [1]. Although susceptibility to dieback is widespread in romaine and leaf lettuces, modern iceberg-type cultivars remain completely free of symptoms when grown in infested soil [1,6]. It appears that the resistance observed in iceberg cultivars was originally introduced into the iceberg genepool from the cultivar Imperial around 70 years ago [3,7]. If true, this suggests that the resistance is effective and highly durable despite extensive cultivation of iceberg cultivars. Through use of molecular marker technology, the single dominant gene (*Tvr1*), which is responsible for the dieback resistance in iceberg lettuce, has been mapped to chromosomal linkage group 2 [1]. Position of the gene was inferred with AFLP and RAPD markers in a population originating from a cross between the resistant cultivar Salinas and the susceptible cultivar Iceberg (cv. Iceberg is a Batavia type lettuce). Another dieback resistance gene was discovered in the primitive romaine-like accession PI491224 [6]. Analysis of resistance in offspring originating from a cross between the two resistant genotypes (Salinas × PI491224) indicates that the resistance locus in PI491224 is either allelic or linked to *Tvr1 *[1]. Because of the increased interest in non-iceberg types of lettuce, introgressing *Tvr1 *into romaine, leaf, and other susceptible types is of high priority for the lettuce industry. However, the breeding process is slow and labor intensive due to a need for extensive field-based testing. Application of marker-assisted selection (MAS) can reduce the need for field screening and accelerate development of dieback resistant material.

To pinpoint the location of the *Tvr1 *gene and develop markers for marker-assisted selection, we employed a combination of classical linkage and association mapping techniques [8]. The association mapping approach is based on the extent of linkage disequilibrium observed in a set of accessions that are not closely related. In contrast to linkage mapping, association mapping is a method that detects relationships between phenotypic variation and genetic polymorphism in existing germplasm, without development of mapping populations. This method incorporates the effects of recombination occurring in many past generations into a single analysis [9] and is thus complementary to linkage analysis. Association mapping has been successfully applied in mapping resistance genes in several diploid and polyploid plant species (e.g. [10-12]). The main drawback of association mapping is the possibility of false-positive results due to an unrecognized population structure. When the trait of interest is more prevalent in one subpopulation (e.g. dieback resistance in iceberg lettuce) than others, the trait will be associated with any marker allele that is in high frequency in that subpopulation (e.g. [13]). Our previous analysis of population structure with molecular markers revealed that cultivated lettuce is divided into several well-defined subpopulations that correspond approximately to different horticultural types [14,15]. Consequently, traits that are strongly correlated with lettuce types display many false-positive results when population structure is ignored. However, these spurious associations disappear when estimates of population structure are included in the statistical model [15]. Therefore, the best approach for avoiding spurious associations in lettuce association studies is to assess relatedness of accessions with molecular markers and to include this information into the statistical model [15].

In the present study we mapped the *Tvr1 *gene using a combination of linkage and association mapping. High-resolution DNA melting curve analysis (HRM) was used to assess polymorphism in mapping populations and to detect haplotypes associated with the disease resistance. The potential for marker-assisted selection was then validated in the genetic backgrounds present in most common horticultural types of lettuce. Finally, we used SNP markers to assess intra- and inter-locus linkage disequilibrium in the *Tvr1 *region.

## Methods

### Linkage mapping population

Recombinant-inbred lines (RILs) were derived from a cross between an F_1 _of cv. Valmaine (dieback susceptible romaine type) × cv. Salinas 88 and cv. Salinas. Both Salinas and Salinas 88 are iceberg type lettuces resistant to dieback whose appearance and performance is the same, except for reaction to Lettuce mosaic virus (Salinas 88 is resistant). Two hundred and fifty three F_8 _RILs were screened for resistance to dieback in multiple trials and 192 of these RILs were randomly selected for genotyping with molecular markers.

### Association mapping set

A set of 68 cultivars, plant introductions (PI), and breeding lines representing all predominant types of cultivated lettuce was used for association mapping. The set includes 8 Batavia types, 5 butterhead types, 5 iceberg types, 5 Latin types, 9 leaf types, 31 romaine types, and 5 stem types (Table 1). The lettuce accessions were selected from material used in breeding programs, ancestors frequently observed in pedigrees, and newly developed breeding lines. For each horticultural type both dieback resistant and susceptible accessions were selected, with the exception of iceberg lettuce, where only resistant cultivars were available, and the Latin type, where only susceptible cultivars were available.

**Table 1:** List of 200 *L. sativa *accessions used in the association mapping study.

Horticultural Type	Resistant	Susceptible
Batavia	AvonCrisp, Batavia Beaujolais, **Drumhead White Cabbage**, **Express**, **Great Lakes 54**, Imperial, **La Brillante**, River Green	Batavia Blonde A Bord Rouge, Batavia Blonde de Paris, **Batavia Reine des Glaces**, Carnival, Fortessa, Hanson, **Holborn's Standard**, **Iceberg**, **New York**, Progress, Tahoe Red, Webb's Wonderful

Butterhead	**Bibb**, **Cobham Green**, Dark Green Boston, **Margarita**, **Tania**, Verpia	Ancora, Dandie, Encore, Lednicky, Madrilene, **MayKing**, Ninja, Saffier, Tinto, Tom Thumb

Iceberg	Astral, Autumn Gold, Ballade, Barcelona, Bix, Black Velvet, Bounty, Bronco, Bullseye, Calmar, Climax, Coyote, Diamond, Duchesse, Eastern Lakes, Empire, Fimba, Formidana, Glacier, Green Lightening, IceCube, Invader, Lighthouse, Mini Green, Misty Day, Monument, **Pacific**, Primus, Raiders, Red Coach, **Salinas**, Salinas 88, **Sea Green**, **Sharp Shooter**, Sniper, Sureshot, Tiber, **Vanguard**, Winterhaven, Winterselect, Wolverine	

Latin		**Barnwood Gem**, Eruption, **Gallega**, **Little Gem**, **Pavane**, **Sucrine**

Leaf	Alpine, **Cracoviensis**, **Grand Rapids**, PI177418, Pybas Green, **Ruby Ruffles**, **Salad Bowl**, Shining Star, Slobolt, **Two Star**, Waldmann's Green	Australian, Cavarly, Coastal Star BS, Colorado, Deep red, Deer's Tongue, Flame, **Lolla Rossa**, **Merlot**, **North Star**, Oak Leaf, Prizehead, Red Oak Leaf, Red Salad Bowl, Red Tide, Redina, Royal Red, Ruby, Squadron, Triple Red, Ventana, Vulcan, Xena

Oil	PI250020, PI251245	

Romaine	**01-778M**, **01-781M**, **01-789M**, Athena, **Bandit**, **Blonde Lente a Monter**, **Defender**, PI171666, PI491209, **PI491214**, PI491224, **Skyway**, Sturgis, **Sx08-003**, **Sx08-004**, **Sx08-005**, **Sx08-006**, **Sx08-007**, **Sx08-008**, **Triple Threat**	Annapolis, Apache, Ballon, Bautista, Brave Heart, Caesar, Camino Real, Chicon des Charentes, **Clemente**, Coastal Star WS, Conquistador, Dark Green Cos, **Darkland**, Eiffel Tower, **Gladiator**, **Gorilla**, **Green Forest**, **Green Towers**, **Heart's Delight**, Infantry, **King Henry**, Larga Rubia, **Lobjoits**, Majestic Red, Medallion, Outback, **Paris White**, **Parris Island Cos**, PI140395, PI169510, PI177426, PI179297, PI220665, PI268405, PI269503, PI269504, PI289064, PI358027, PI370473, PI420389, Queen of Hearts, **Reuben's Red**, Romaine Chicon, Rouge d'Hiver, **Short Guzmaine**, Signal, **Tall Guzmaine**, Triton, Ultegra, Valcos, **Valmaine**, Wayahead, **White Paris**

Stem	Balady Bahera, **Balady Banha**, **Balady Barrage**, **Celtuce**, Chima	**Balady Aswan**, Balady Cairo, **PI207490**

Accessions that were sequenced are in bold; the remaining accessions were analyzed with the HRM approach only.

### Validation set

To validate the marker-trait association detected in the association mapping set, a validation set of 132 accessions was screened for disease resistance and genotyped with the marker, Cntg10192. This set represents the spectrum of phenotypic and genotypic variability observed in cultivated lettuce and includes 12 Batavia types, 11 butterhead types, 36 iceberg types, 1 Latin type, 25 leaf types, 2 oil types, 42 romaine types, and 3 stem types (Table 1).

### Assessment of dieback resistance

Dieback resistance data were obtained from field observations as previously described [15]. Susceptibility was evaluated by seeding lettuce directly in the field in Salinas, CA, from which TBSV and LNSV had previously been isolated from plants exhibiting characteristic dieback symptoms [1]. The experiment was comprised of two complete blocks, with ~30 plants per genotype per block. Plants were seeded in two rows on 1 m wide beds and were thinned to obtain spacing of 30 cm between plants. Standard commercial practices were used for irrigation, fertilization, and pest control. Plants were checked weekly for disease symptoms in order to discriminate between plants dying due to dieback and those due to unrelated causes. The percentage of plants that showed typical dieback symptoms (or were dead due to dieback) was recorded at harvest maturity. Accessions with < 5% of symptomatic plants were considered to be resistant. To minimize the possibility of inaccurate scoring, all accessions were tested in at least three independent field trials. If results from all three trials were consistent, the material was not tested further. In the case of inconsistent results, material was retested in another two independent trials, after which all accessions were classified into one of the two groups. The resistance and susceptibility classification was subsequently used in statistical analyses.

### DNA isolation

Tissue from young leaves of about one-month-old plants was collected and immediately lyophilized. Lyophilized samples were ground to fine powder using a TissueLyser mill (Qiagen, Valencia, CA), before extracting genomic DNA with the NucleoSpin Plant II kit (Macherey-Nagel, Betlehem, PA). The DNA concentration and quality was analyzed with an ND-1000 Spectrometer (NanoDrop Technologies, Wilmington, DE) and gel electrophoresis.

### Polymerase chain reaction, allele detection, and product sequencing

Primer pairs were designed for each marker from EST (expressed sequence tag) sequence with the PRIMER 3 software [16]. The selection of ESTs from the CGPDB database [17] was based on their position in the genome - only ESTs previously mapped to the linkage group 2 were considered for development of markers. Due to the presence of introns in genomic DNA, primers for several markers had to be designed more than once to obtain an amplicon for the given marker. The polymerase chain reaction (PCR) was performed in a 20 μl volume containing 10 ng of genomic DNA as a template, 200 μmol/L of each dNTP, 1× Standard *Taq *PCR buffer with 1.5 mmol/L MgCl_2_, 1.2 U *Taq *polymerase (all from New England Biolabs, Ipswich, MA), and forward and reverse primers at a concentration of 0.25 μmol/L each. The reaction conditions were as follows: 95° for 2 min, followed by 35 cycles of 95° for 30 s, annealing temperature (Table 2) for 30 s, and 72° for 30 s, with final extension of 72° for 5 min. Amplification was performed in an MJ Research Tetrad Thermal Cycler (MJ Research, Waltham, MA). The PCR products were analyzed on gels composed of 0.7% agarose (Fisher Scientific, Pittsburgh, PA) and 1.15% Synergel (Diversified Biotech, Boston, MA) run with 0.5× TBE buffer. PCR samples were stained prior to electrophoresis with 1× GelRed (Biotium, Hayward, CA). Alternatively, the PCR products were separated using an HDA-GT12 DNA analyzer and scored by Biocalculator software (both from eGene, Irvine, CA). If sequencing was needed, PCR products were first treated with Exonuclease I and subsequently with Antarctic Phosphatase (both from New England Biolabs). DNA sequencing was performed using ABI BigDye Terminator (v3.1; Applied Biosystems, Foster City, CA) according to the manufacturer's protocol, except that 5-μl reactions were performed with 0.25 μl of BigDye on an ABI 3730xl DNA sequencing machine with 50 cm arrays.

**Table 2:** Information for nine markers that were sequenced from a set of 68 *L. sativa *accessions.

Marker	EST/Contig in CGPDB	Primers (5' - 3')	Ta (°C)	Mg (mM)	Amplicon size (bp)
LK1457	QG_CA_Contig4638	F - AGGAGCAAAGGAAAGGCTTC	57	1.5	636-648
		R - TGCAACTTCTTCAGCCAATG			
Cntg10044	CLS_S3_Contig10044	F - GCATGCCGATTACTCCTTTC R - TCCCCAATCACCTAAGATGG	57	1.5	845-860
QGG19E03	QGG19E03.yg.ab1	F - ATATCCCACCGCCCATAGAT	57	1.5	711-720
		R - ACGCAACTAACCCGTTTCAT			
Cntg4252	CLS_S3_Contig4252	F - GGGGAGTTCAGACGTTCAGT	57	1.5	1160
		R - CGAATTGATACACCGCAAAA			
Cntg10192	CLS_S3_Contig10192	F - CTCGTTTTCAACACCGACAA	57	1.5	349
		R - TTGTCTCCGGCACTGTATCATCG			
CLSM9959	CLSM9959.b1_N18.ab1	F - TGCTCAATTACACTCGAACCA	57	1.5	326
		R - CTTCATGGAGAGAAATACAAGGTC			
CLSZ1525	CLSZ1525.b1_J22.ab1	F - TTGTTGAAATTATAAACACGAAGCA	57	3	499-629
		R - CAACAAAGGATGTCTCAAATTCA			
QGC11N03	QGC11N03.yg.ab1	F - GCACCTGATGGCTGAATATG	57	1.5	569-581
		R - CATCCTCAATCGCTTGTGTT			
Cntg11275	CLS_S3_Contig11275	F - GGAGAAATTTTGGAGCTGTAATTAC	61	1.5	765-956
		R - GGAGGTATGTTGAGGTACATGAC			

Columns indicate marker name, EST or Contig information in the CGPDB database, forward and reverse primers, annealing temperature (Ta), magnesium concentration in PCR reaction, and size of amplicon. Marker QGG19E03 could not be successfully amplified from 13 accessions even though 34 primer combinations were tested.

DNA sequences were analyzed with CodonCode Aligner v. 2.0.6 (CodonCode Corporation, Dedham, MA). We detected three types of polymorphism in our sequences - single feature polymorphism (SFP), insertions and deletions (indels) and variable number tandem repeats (VNTRs). Most of the SFPs that had been detected using the Affymetrix GeneChip [17] were due to a single nucleotide polymorphism (SNP), but in five cases due to a single base indel. Since Haploview cannot handle missing values, missing bases were substituted prior to data analysis with an appropriate single nucleotide. Because all single-base indels could be tagged with SNPs from the same marker locus (as described below), we use the term SNP throughout the text. Both indels and VNTRs were excluded from data analysis, unless otherwise noted in the text.

### High-resolution DNA melting curve (HRM) analysis

EST-derived markers were screened for polymorphism using high-resolution melting curve analysis. Primer pairs for each marker were developed with the PRIMER 3 software and tested for optimal amplification using a temperature gradient (from 58-67°). Amplifications were performed in 10 μl reactions containing 10 ng DNA, 200 μmol/L of each dNTP, 0.6 U *Taq *polymerase, 1× Standard *Taq *buffer with 1.5 mmol/L MgCl_2 _(all from New England Biolab), 1× LCGreen Plus Melting Dye (Idaho Technology, Salt Lake City, UT), 0.25 μmol/L of each primer, and 15 μL of mineral oil (USB Corporation, Cleveland, OH). PCR was performed on a MJ Research Tetrad Thermal Cycler with an initial denaturation of 95° for 2 min, followed by 45 cycles of 95° for 30 s, annealing temperature (Table 3) for 30 s, and 72° for 30 s, with final extension of 72° for 5 min. To facilitate heteroduplex formation samples were subjected, after the final extension, to 95° for 30 s followed by cooling to 25° for 30 s. Simulation of a heterozygote was achieved by mixing equal amounts of DNA from the two parental homozygous cultivars before PCR amplification. Melting-curve analysis was performed in a 96-well plate (HSP-9665, Biorad, Hercules, CA) on a LightScanner System and with the LightScanner software v. 2.0.0.1331 (both from Idaho Technology). Melting curves were analyzed as described in the LightScanner software manual.

**Table 3:** Information for six markers that were analyzed in the (Valmaine × Salinas 88) × Salinas mapping population with the HRM approach.

Marker	EST/Contig in CGPDB	Primer (5' - 3')	Ta (°C)	Mg (mM)	Amplicon size (bp)
LK1457	QG_CA_Contig4638	F - AGGAGCAAAGGAAAGGCTTC	64	3	636-648
		R - TGCAACTTCTTCAGCCAATG			
Cntg4252	CLS_S3_Contig4252	F - AGAACCAGGTCGAATCATGG	61	1.5	208
		R - TTCTCGCCGTTGAGAAGAAT			
		Probe - AAGTGGCTATACAGCTTTGATCATAACGA			
Cntg10192	CLS_S3_Contig10192	F - CTCGTTTTCAACACCGACAA	61	1.5	185
		R - TAGGTGGGTCCGACTTTGAG			
CLSM9959	CLSM9959.b1_N18.ab1	F - TGCTCAATTACACTCGAACCA	61	1.5	326
		R - CTTCATGGAGAGAAATACAAGGTC			
CLSZ1525	CLSZ1525.b1_J22.ab1	F - GAAGAAACTCATGAATCTGCTCAA	62	3	157-158
		R - TTTGCTCAAGAACTCTTAAACCATT			
Cntg11275	CLS_S3_Contig11275	F - CCAAACCATAGGGACGAAAA	61	1.5	252-260
		R - GGAGGTATGTTGAGGTACATGAC			

Marker Cntg4252 was analyzed in combination with a probe. Polymorphisms for three markers that are not shown in the table were detected by electrophoresis. All information for these is the same as in Table 2.

### Linkage mapping

One hundred and ninety two RILs derived from a cross between an F_1 _of cv. Valmaine × cv. Salinas 88 and cv. Salinas were genotyped with EST-derived markers. Selection of markers for this first round of genotyping was based on the molecular linkage map developed from an interspecific cross between *L. sativa *cv. Salinas and *Lactuca serriola *accession UC96US23 [17,18]. Twenty markers were selected to evenly cover linkage group 2 in intervals of approximately 10 to 20 cM. After preliminary mapping of the resistance gene, the region containing *Tvr1 *was saturated with markers originating from a microarray-based study also carried out on the Salinas × UC96US23 population [17]. Marker polymorphism was tested with HRM analysis, unless the difference between segregating alleles could be visually observed using gel electrophoresis. If polymorphism could not be observed with HRM analysis, PCR products from the two parental genotypes were sequenced and new primers were designed for HRM. Statistical analysis of the linkage between molecular markers and dieback resistance was performed by MapManager QTX software [19]. Dieback resistance for each RIL was considered as a bi-allelic qualitative trait (resistant or susceptible) and used for linkage analysis.

### Association mapping and assessment of population structure

Association mapping was performed on a set of 68 accessions from seven horticultural types of lettuce (Table 1). In the first step, markers closely linked to the *Tvr1 *gene were amplified from each accession and sequenced. In the second step, the sequenced amplicons were analyzed for polymorphism with the CodonCode software and inputted into Haploview v. 4.2 [20]. Intra-locus SNPs were tagged in Haploview with the Tagger function at *r*^2 ^= 1. Untagged SNPs from all markers and a representative SNP for each tag were then entered into TASSEL v. 2.0.1 [21]. TASSEL was subsequently used to test for association between individual SNPs and resistance to dieback while accounting for the population structure. Both *p*-values for each SNP and percent of phenotypic variation explained by the model (*R*^2^) were calculated with TASSEL after 100,000 permutations.

Prior to association analysis, the population structure in the set of 68 accessions was assessed with thirty EST-SSR markers distributed throughout the genome [14] using the computer program STRUCTURE 2.2 [22]. Ten runs of STRUCTURE were done by setting the number of populations (*K*) from 1 to 15. For each run, the number of iterations and burn-in period iterations were both set to 200,000. The *ad hoc *statistic [23] was used to estimate the number of subpopulations. The optimum number of subpopulations (*K *= 5) was subsequently used to calculate the fraction of each individual's genome (*q*_*k*_) that originates from each of the five subpopulations. The *q*_*k *_values obtained from STRUCTURE were used as covariates in the statistical model given by TASSEL.

### Genetic variation and a linkage disequilibrium estimate

The level of genetic variation at the nucleotide level was estimated as nucleotide polymorphism (*θ*, [24]) and nucleotide diversity (*π*, [25]). To test the neutrality of mutations, we employed Tajima's D test [26], which is based on differences between *π *and *θ*. Analyses of genetic variation and estimates of haplotype diversity (Hd) were carried out using DnaSP v. 5.00.04 software [27].

Linkage disequilibrium (*r*^2^) between pairs of SNP loci in the genome was calculated with Haploview and the values were pooled over the entire data set. Decay of LD with distance was estimated using a logarithmic trend line that was fitted to the data. Distances between markers were calculated from their respective positions on the consensus molecular linkage map. The consensus map was created with JoinMap v. 2.0 [28] from the Salinas × UC96US23 map [18] and the (Valmaine × Salinas 88) × Salinas map (present work). SNPs with frequency < 5% were excluded from the analysis.

## Results

### Linkage mapping

**Figure 2:**
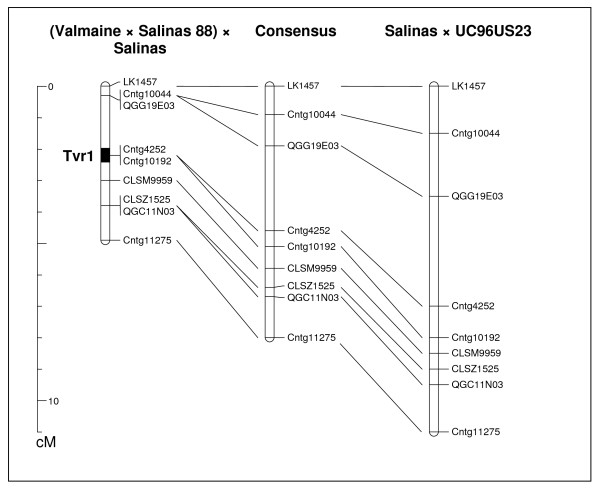
**Part of chromosomal linkage group 2, showing nine markers linked to the *Tvr1 *gene**. The map on the left is based on segregation observed in the (Valmaine × Salinas 88) × Salinas population, the map on the right is based on segregation observed in the Salinas × UC96US23 population, and the map in the center is a consensus map developed from the two linkage maps. A black bar on the (Valmaine × Salinas 88) × Salinas map indicates the estimated position of the *Tvr1 *gene.

Cv. Salinas was resistant, while cv. Valmaine was susceptible to dieback in seven trials over four years. The disease index for cultivar Salinas ranged from 0% to 2% and for cultivar Valmaine from 69% to 100% among these field experiments. We found highly significant correlations (from *r *= 0.63 to *r *= 0.89, *p *< 0.001) between estimated percentages of symptomatic plants in independent trials (data not shown). From 253 RILs tested in multiple experiments, 124 were resistant and 129 were susceptible. This segregation is not significantly different from the expected 1:1 ratio, consistent with a single gene effect. The segregation ratio in the 192 individuals that were used for mapping of the resistance gene was 92 resistant to 100 susceptible. Linkage mapping on the framework map with markers spaced about 10 cM to 20 cM apart indicated that the *Tvr1 *gene is linked to the marker LK1457. When this genomic region was saturated with additional markers, the *Tvr1 *locus co-segregated with two of them. These two markers are based on ESTs Cntg4252 and Cntg10192. Besides the two co-segregating markers; another six markers were located within 5 cM of the resistance gene. These markers are based on ESTs Cntg10044, QGG19E03, CLSM9959, CLSZ1525, QGC11N03, and Cntg11275 (Figure 2).

### Nucleotide polymorphism

The nine markers closely linked to *Tvr1 *were amplified and sequenced from a set of 68 accessions. This set included all major horticultural types of lettuce that had been previously screened for resistance to dieback. Thirty-six of the accessions showed resistance to the disease and 32 were susceptible. Five of the seven horticultural types included both resistant and susceptible genotypes. The two exceptions were iceberg and Latin types, where only resistant and susceptible accessions respectively were available. Sequencing of over 370 kb from nine markers in the 68 accessions revealed 160 SNPs, six indels (3 bp to 12 bp long), and two VNTRs (in markers CLSZ1525 and Cntg11275). Sequenced markers were between ~300 bp to 1 kb long, having 3 to 35 polymorphic sites, and 3 to 10 haplotypes (Table 4). Haplotype diversity (Hd) was similar in all markers and ranged from 0.593 to 0.809. Values for nucleotide diversity (*π*) ranged from 2.37 × 10^-3 ^to 8.67 × 10^-3 ^(exon and intron values combined) with an exception of marker CLSZ1525 that had a value of 31.22 × 10^-3^. Nucleotide polymorphism (*θ*) was in the range from 1.54 × 10^-3 ^to 8.30 × 10^-3^. However, two markers each had a level of polymorphism above 10 × 10^-3^; marker QGC11N03 (11.32 × 10^-3^) and marker CLCZ1525 (15.23 × 10^-3^). Since the sequenced regions of markers LK1457, Cntg10044, Cntg4252, and Cntg11275 contain both introns and exons, it is possible to compare polymorphism between the two groups. While there was no significant difference in haplotype diversity between introns and exons, both nucleotide diversity (*π*) and polymorphism (*θ*) were approximately 4.7 fold higher in introns (*p *= 0.01998 for *π*, *p *= 0.00018 for *θ*) (data not shown). Values of Tajima's *D *ranged from -1.224 to 3.397. Significant values of this parameter were calculated for markers LK1475, Cntg4252, and Cntg11275 when combined intron and exon data were considered and for markers Cntg10192, CLSM9959, and CLSZ1525 that contain exons only.

**Table 4:** Estimates of nucleotide variation in nine markers linked to the *Tvr1 *gene.

Marker	Size (bp)	Polymorphic sites (S)	Haplotypes	Haplotype diversity (Hd)	Nucleotide diversity (*π *× 10^-3^)	Nucleotide poly-morphism (*θ *× 10^-3^)	Tajima's *D*
LK1457	526	12	5	0.705	8.47	4.75	2.19978 *
LK1457 (exons)	270	2	3	0.634	3.02	1.54	1.59391
Cntg10044	727	29	10	0.760	5.10	8.30	-1.22415
Cntg10044 (exons)	330	6	8	0.758	4.55	3.78	0.4853
QGG19E03 (exons)	673	14	5	0.593	7.05	4.98	1.27593
Cntg4252	1021	16	6	0.747	5.91	3.29	2.3525 *
Cntg4252 (exons)	852	7	5	0.722	2.37	1.73	0.9381
Cntg10192 (exons)	348	3	3	0.644	5.33	2.39	2.78285 **
CLSM9959 (exons)	302	4	5	0.763	6.27	2.75	2.7119 **
CLSZ1525 (exons)	492	35	5	0.783	31.22	15.23	3.3968 ***
QGC11N03 (exons)	518	28	7	0.783	7.43	11.32	-1.09141
Cntg11275	840	29	7	0.809	8.67	4.67	2.11930 *
Cntg11275 (exons)	384	4	5	0.729	2.55	1.65	1.23874

Five of the markers consist of exons only, while the remaining four markers consist of a combination of exons and introns. Analyzed fragments are shorter than amplified markers, because indels, VNTRs, and some poor sequences were deleted prior to data analysis. *, **, and *** indicate the significance of Tajima's *D *test at *p *≤ 0.05, *p *≤ 0.01, and *p *≤ 0.001 (respectively).

### Association mapping

Evaluation of population structure in a set of 68 accessions revealed that the best estimate of the number of subpopulations was five (*K *= 5) (data not shown). These subpopulations corresponded approximately with the horticultural types. Best separated were crisphead (this type combines iceberg and Batavia), romaine, butterhead plus Latin, and stem-type lettuces. Leaf-type lettuce was not separated in a single sub-population. From 160 SNPs that were identified in the nine markers closely linked to the *Tvr1 *gene, 60 were non-redundant for discrimination of haplotypes. These unique SNPs were included together with the estimates of population structure in the association analysis performed with TASSEL. Eighteen SNPs, one indel, and one VNTR were significantly (*p *≤ 0.001) associated with the resistance allele (Table 5). Significant SNPs were detected on all markers with the exception of marker Cntg4252, for which the best value was *p *= 0.0042. The SNP with the largest effect was found on marker Cntg10192 at position 72. This SNP matches perfectly with the observed resistance (*R*^2 ^= 100%). An additional SNP from the same tag is located at position 54. Both of these SNPs have C ⇔ T base substitutions where T is associated with resistance and C with susceptibility to dieback. Although both mutations are located in the coding region, they are synonymous and do not lead to changes in amino acids.

**Table 5:** Association between SNPs and dieback resistance in a set of 68 *L. sativa *accessions.

Marker	SNP position	*p*-value	R^2 ^%	Tagged SNPs
LK1457	137	0.00008	29.2	513
	224	0.00001	48.7	235, 236, 251
	318	0.00037	25.9	482

Cntg10044	9	0.00470	19.4	
	27	0.00022	24.8	
	109*	0.00001	32.3	
	170**	0.00300	20.1	
	337	0.00085	22.5	
	733	0.00001	33.9	

QGG19E03	27	0.00001	53.9	46, 525, 574, 594
	355	0.00130	33.0	393, 415, 480, 597, 598

Cntg4252	472	0.00420	22.6	480, 486, 489, 490. 492, 493, 499, 544, 577

Cntg10192	72	0.00001	100.0	54
	100	0.00001	40.9	

CLSM9959	77	0.00001	38.0	
	242	0.00210	22.7	

CLSZ1525	84	0.00498	19.4	100, 102, 144, 236, 250, 258, 279, 309, 399, 400, 402, 457, 464, 483
	89	0.00001	48.6	107, 110, 116, 123, 149, 181, 296
	465	0.00001	33.2	
	VNTR***	0.00001	48.8	

QGC11N03	42	0.00010	29.8	
	50	0.00001	45.0	
	448	0.00001	50.4	

Cntg11275	7	0.00001	42.5	
	431	0.00001	38.0	525, 534, 559, 583, 590, 748, 798, 799
	623	0.00031	27.4	661, 685, 742, 766, 767

Columns indicate markers, SNP position in the marker, the *p*-value of association, the percent of phenotypic variation explained by the SNP (R^2 ^%), and SNPs from the same tag. SNPs with a *p*-value of ≤ 0.005 are shown, but only those with *p *≤ 0.001 are considered to be significant. *, **, and *** denote indel, SFP, and Variable Number of Tandem Repeats (respectively).

### Linkage disequilibrium

**Figure 3:**
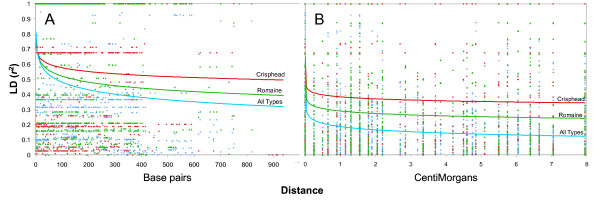
**Decay of linkage disequilibrium (*r*^2^) as a function of distance between two SNPs**. Pooled data from nine markers in the *Tvr1 *region were used to estimate the (A) intra-locus and (B) inter-locus linkage disequilibrium. Distances for the intra-locus LD are in base-pairs (bp), while those for inter-locus LD are in centimorgans (cM). The lines indicate logarithmic curves fitted to the data from a set of 68 accessions representing either all horticultural types, or data from crisphead or romaine types only.

Intra- and inter-locus LD were analyzed on nine markers flanking the *Tvr1 *gene. Intra-locus LD shows a gradual decline as a function of distance and was estimated to have a value of *r*^2^~0.322 at 900 bp (Figure 3). To observe inter-locus LD, we calculated *r*^2 ^between SNPs detected in different markers. Analysis showed progressive, but slow, decay of LD and SNPs separated by ~8 cM had an *r*^2 ^value of 0.124. Since estimates of LD can be substantially affected by a population structure, we calculated LD decay in two well-defined subpopulations with sufficient numbers of individuals (romaine and crisphead). Estimated values of *r*^2 ^at 900 bp were 0.396 and 0.498 for romaine and crisphead types, respectively. Similarly, at a distance of ~8 cM we observed a larger LD in both types (*r*^2 ^0.247 for romaine, and 0.345 for crisphead) than in the whole set that combined multiple subpopulations.

### Development of markers for marker-assisted selection

**Figure 4:**
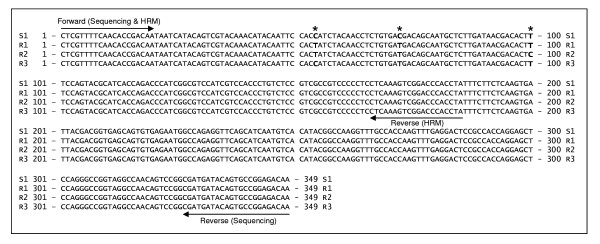
**Sequence comparison of the four haplotypes detected at marker Cntg10192**. Three haplotypes (R1, R2, & R3) are associated with dieback resistance while the S1 haplotype is always associated with susceptibility to the disease. Horizontal arrows indicate positions of the primers used for sequencing and for the HRM analysis. Asterisks show the positions of the three SNPs present in the marker.

**Figure 5:**
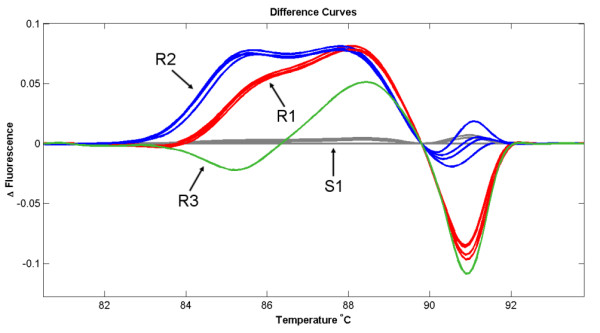
**The differences in shapes of melting curves illustrate the detection of four homoduplexes corresponding to haplotypes R1, R2, R3, and S1**. For example, the haplotype R1 was detected in iceberg cv. Salinas, haplotype R2 in primitive romaine-like accession PI491224, R3 in *L. serriola *accession UC96US23, and S1 in romaine cv. Valmaine.

The resistance-SNP association observed in the set of 68 accessions was detected through sequencing of PCR amplicons from individual accessions. In order to accelerate and simplify the test of association, we developed a primer pair that allowed detection of polymorphism in the marker Cntg10192 through high-resolution melting analysis. These primers amplify a 185 bp product that contains all three SNPs detected in the marker Cntg10192 at the positions 54, 72, and 100. The first two SNPs match perfectly with the resistance allele, while the third SNP explains 40.9% of the trait variation. As with the first two SNPs, the third SNP has a C ⇔ T substitution. All susceptible genotypes carry the T allele, while resistant genotypes have either the T or C alleles at the third SNP. It appears that the T allele in the resistant material is associated with the resistance present in cv. Salinas and most of the other iceberg cultivars, whereas the C allele is associated with the resistance present in the three lines (01-778 M, 01-781 M, 01-789 M) that originate from the romaine-like primitive accession PI491224. Marker Cntg10192, therefore, not only allows for the detection of alleles associated with dieback resistance, but also separates alleles of different origins. To further investigate polymorphism in this genomic region we sequenced two accessions from *L. serriola*, a wild species closely related to cultivated lettuce. One of the accessions (UC96US23) is resistant to the disease, while the other one (PI274808) is susceptible. The susceptible genotype has the same allele sequence as all susceptible *L. sativa *accessions. The resistant accession has a haplotype similar to cv. Salinas but instead of the T allele at position 54, it carries the C allele. The three SNPs at the marker Cntg10192 can thus distinguish four different haplotypes; three resistant and one associated with susceptibility (Figure 4). Haplotype R1 (cv. Salinas) has the T-T-T allele combination at positions 54, 72, and 100. Haplotype R2 (PI491224) carries the T-T-C combination, while haplotype R3 (UC96US23) carries the C-T-T alleles. Disease susceptibility was always associated with the S1 haplotype (cv. Valmaine) that carries the C-C-T combination. All four haplotypes can easily be separated through high-resolution melting analysis (Figure 5).

### Marker validation

Validation of the haplotype-resistance association detected in the set of 68 *L. sativa *accessions and two *L. serriola *genotypes was performed on an additional set consisting of 132 accessions of *L. sativa*. This set also contained diverse material that represented a broad spectrum of the variability present in cultivated lettuce. We used the HRM approach for marker Cntg10192 and, as before, all genotypes that were susceptible to the disease carried haplotype S1, while resistant material had either the R1 or R2 haplotypes (Figure 5). This association was independent from population structure and was observed across all horticultural types.

## Discussion

### Nucleotide polymorphism

Nucleotide polymorphism was observed in all nine markers that were sequenced from the region flanking the *Tvr1 *gene. The rate of nucleotide substitutions in a set of 68 accessions translates into ~1 SNP per 149 bp (1/*θ*) between pairs of randomly selected sequences. This SNP frequency was somewhat lower when only coding regions were considered (1 SNP per 187 bp). These values are well within the range observed for other plant species. For example, the average SNP frequency is 60 bp in aspen (*Populus tremula *L.) [29], 87 bp in potato (*Solanum tuberosum *L.) [30], 104 bp in maize (*Zea mays *L.) [31], 130 bp in sugar beet (*Beta vulgaris *L.) [32], 232 bp in rice (*Oryza sativa *L.) [33], 435 bp in sorghum (*Sorghum bicolor *L.) [34], 585 bp in tomato (*Solanum lycopersicum *L.) [35], and 1030 bp in soybean (*Glycine max *L.) [36]. Both nucleotide polymorphism (*θ *= 6.7 × 10^-3^, in the coding region 5.4 × 10^-3^) and nucleotide diversity (*π *= 9.6 × 10^-3^, in the coding 8.0 × 10^-3^) of lettuce are similar to that observed in maize (*θ *= 9.6 × 10^-3^, *π *= 6.3 × 10^-3^), potato (*θ *= 11.5 × 10^-3^, *π *= 14.6 × 10^-3^), and sugar beet (*π *= 7.6 × 10^-3^), but larger than in tomato (*θ *= 1.71 × 10^-3^, *π *= 1.34 × 10^-3^), and soybean (*θ *= 0.97 × 10^-3^, *π *= 1.25 × 10^-3^) [30-32,35-37]. If results from the analyzed region correspond to those for the whole genome, sequence variation in lettuce is relatively high for a selfing species. It was previously observed that selfing species have generally lower levels of sequence variation than outcrossing species because of smaller effective population sizes [38]. Although polymorphism in lettuce appears to be considerably larger than in selfing soybean and tomato, it is similar to that observed in rice, which is also a self-pollinating species. The ratio of nucleotide diversity in coding (exon) and non-coding (intron) sequences was not analyzed in detail, since data from only four markers (LK1457, Cntg10044, Cntg4252, Cntg11275) are available. However, the ratio (0.32) across these markers appears to be smaller than in Arabidopsis (*Arabidopsis thaliana*) (0.38 [36]), soybean (0.45 [36]), maize (0.65 [31]), and potato (0.71 [30]). This difference is likely due to a higher level of functional constraint on the perigenic sequence [36] of lettuce. Measures of haplotype diversity (Hd) were based on estimated haplotype frequencies [39], and calculated using the DNAsp software. This measure of diversity is analogous to the heterozygosity at a single locus, and is at its maximum when haplotypes observed in the sample occur at equal frequencies [40]. Diversity based on haplotypes ranged from 0.593 in QGG19E03 to 0.809 in marker Cntg11275, with an average value of 0.732 ± 0.024. These values are higher than in rice (0.507 ± 0.048 [41]), soybean (0.52 [36]), and human (0.651 ± 0.016 [40]). It is possible that the high level of diversity is related to the way that selection of the 68 accessions was performed. We included dieback resistant and susceptible material from all predominant horticultural types, thereby selecting haplotypes at similar frequencies. It would be interesting to observe how haplotype diversity changes in different genomic regions and/or for a different set of accessions.

To test the neutrality of mutations, Tajima's *D *was calculated for all surveyed markers. The average *D *(1.48 ± 0.45 for the coding regions and 1.61 ± 0.56 for whole fragments) was larger than in soybean (1.08 [36]), potato (0.5 [30]), and sorghum (0.30 [34]). A positive *D *value indicates a deficit of low-frequency alleles relative to what is expected. Since large *D *values can be caused by a population subdivision [37], it is possible that the presence of subpopulations in the analyzed set of lettuce accessions affects both haplotype diversity and the *D *values. When neutrality of mutations was tested in individual markers, three markers closely linked to the *Tvr1 *gene (< 1.5 cM) had Tajima's *D *values significantly higher (*p *≤ 0.01) than expected (Cntg10192 - 2.78, CLSM9959 - 2.72, CLSZ1525 - 3.40). Again, the population structure or selection at the *Tvr1 *locus or the marker itself could have caused departures from neutrality.

### Linkage disequilibrium

The decay of LD for the *Trv1 *region was relatively slow when measured both within individual markers and between markers flanking *Tvr1*. Estimated values of *r*^2 ^were ~0.322 at 900 bp, and ~0.124 at 8 cM. A fitted logarithmic curve shows that the *r*^2 ^value of 0.2 (often considered the threshold for estimating the extent of LD) is reached somewhere between 0.5 cM to 1 cM. LD of SNP markers observed in some other selfing species was similar; LD in Arabidopsis was 250 kb or 1 cM [42]) and in soybean was ~50 kb [36]). Intra-locus LD decayed very little in tomato, with the log trend showing *r*^2 ^> 0.6 at 900 bp [35]. However, it is problematic to compare decay of LD across species due to the large variability in LD quantification. LD depends on a combination of many factors, such as the origin of the population, selected set of accessions, analyzed genomic region, molecular marker system, and presence of unidentified subpopulations. Hyten [43] compared four different soybean populations for levels of LD decline. While in the domesticated Asian *G. max *population LD did not decline along the 500 kb sequenced region, the wild *Glycine soja *population had a large LD decline within the LD block size averaging 12 kb. Comparable observations were not only made in the selfing Arabidopsis [44], but also in the outcrossing maize [31] and aspen [29]. Our results show a large difference between estimates of LD when analyses were performed across all horticultural types or within each individual type. While the estimate of *r*^2 ^at a distance of 8 cM was 0.124 for the whole set, it was 0.247 for romaine type and 0.345 for crisphead lettuce. Because only a relatively small part of the genome was analyzed in the present work, it is not possible to calculate LD at distances over 8 cM. However, the trend for the logarithmic curve suggests that LD could reach more than 15 cM in romaine and probably more than 25 cM in crisphead types before declining to the value of *r*^2 ^< 0.2. When only iceberg types (a subtype of crisphead) were included in the analysis, LD was still at its maximum (*r*^2 ^= 1) at a distance of 8 cM (data not shown). Although these observations come from a limited number of individuals, they are supported by the fact that the modern iceberg-type lettuce has an extremely limited genetic diversity [14,15] that is frequently associated with extensive LD.

### Linkage mapping

A previous study on the Salinas × Iceberg mapping population showed that the single, dominant gene (*Tvr1*) located on linkage group 2 confers resistance to lettuce dieback [1]. We confirmed that the gene is located on linkage group 2 and pinpointed its position with markers Cntg4252 and Cntg10192. Both of these markers co-segregated with the resistance allele in 192 RILs derived from the (Valmane × Salinas 88) × Salinas cross. The molecular linkage map based on the (Valmane × Salinas 88) × Salinas cross showed good colinearity in order of the markers with the map based on the interspecific cross between cv. Salinas and *L. serriola *accessions UC96US23 [18]. However, the interval from LK1457 to Cntg11275 is more than twice the size when estimated from the interspecific cross (11.0 cM and 4.9 cM, respectively). Similarly, while markers Cntg4252 and Cntg10192 co-segregate in the intraspecific map, they are separated by 1 cM on the interspecific map, despite the latter being based on fewer RILs. These values are within the range of other observations on intra- and interspecific maps of lettuce [45]. Colinearity between the two maps allows for development of a consensus map that places markers Cntg4252 and Cntg10192 0.5 cM apart.

### Association mapping

We identified the genomic region carrying resistance against dieback and nine markers closely linked with the *Tvr1 *gene through linkage analysis. We subsequently used this information to test the linked markers for association with the disease resistance on a set of 68 diverse accessions. Eight of the nine markers showed highly significant association with dieback resistance, consistent with the *Tvr1 *gene being located in this region. Although the threshold for declaring association significant was set at *p *< 0.001, most of the associations were significant at *p *≤ 0.00001. The only exception was marker Cntg4252, where the most significant association reached only *p *= 0.0042. The low association between SNPs at this marker and dieback resistance was somewhat unexpected, since Cntg4252 co-segregated with the resistance allele in the (Valmaine × Salinas 88) × Salinas mapping population. While unexpected, it is not uncommon that markers closely linked with a trait in a mapping population do not show association when tested on a set of diverse accessions. This problem is well documented in potato, where markers linked to the *Gro1 *and *H1 *resistance genes in the mapping population were tested on 136 unrelated cultivars. The *Gro1*-specific marker was not correlated with the resistance phenotype, while *H1*-specific marker was indicative of resistance in only four cultivars [46]. A similar example can be shown for lettuce, where markers most tightly linked to the *cor *resistance gene were the least useful for diagnostic when tested in a large collection of cultivars [47]. There are several other examples of markers tightly linked to resistance genes, but whose use present problems in material different from the original in which they were identified [48]. Therefore, an important requirement for any molecular marker used in MAS is not just its applicability in a specific cross, but its association in a wide gene pool.

From SNPs that were significantly associated with dieback resistance, the best fit was observed for those located in marker Cntg10192. This is the second of two markers, the other being Cntg4252, that co-segregated with the resistance allele in the mapping population. It is intriguing that one of the two markers co-segregating with the *Tvr1 *allele in the mapping population showed no significant association in a set of diverse accessions, while the other showed a perfect match. Although these two markers were not separated in the intraspecific population, the linkage map developed from the Salinas × UC96US23 cross indicates that they are 1 cM apart. Therefore it is possible that testing more RILs from the intraspecific population would separate the two markers and *Tvr1*. Association of SNPs from marker Cntg10192 with the resistance allele was validated in a larger set of 132 diverse accessions from several horticultural types. The marker-trait association was observed not only in *L. sativa*, but also in two *L. serriola *accessions included in the study. However, while the susceptible haplotype is identical in both species (S1), the resistant haplotypes are different (R1 & R2 in *L. sativa*, and R3 in *L. serriola*). To investigate the relationship between *Tvr1 *and the resistance observed in *L. serriola*, we screened 119 F_8 _RILs from the Salinas × UC96US23 population for resistance to dieback. If *Tvr1 *and the resistance locus from UC96US23 were distinct and unlinked, approximately 25% susceptible offspring would be observed. However, since all RILs were resistant to the disease (data not shown), we concluded that the resistance locus in UC96US23 is either allelic or linked to *Tvr1*. The same conclusion was reached for the resistance locus in the primitive romaine-type accession PI491224 [1]. The three resistance loci are associated with three distinct haplotypes; resistance in cv. Salinas with R1, in PI491224 with R2, and in UC96US23 with R3.

Even though all 200 *L. sativa *accessions from the two testing sets showed the same haplotype-resistance association, it is unlikely that the EST from which this marker was derived is directly involved in dieback resistance. A search for protein similarity in the NCBI database [49] indicates that Cntg10192 is similar to the copper ion binding protein from castorbean (*Ricinus communis *L., EEF39175.1, similarity 5e^-49^) and the plastocyanin-like domain-containing protein from Arabidopsis (NP_563820, similarity 5e^-43^). The annotated functions of these two proteins do not imply an obvious role in plant-pathogen interactions [50]. Moreover, the two substitutions (at positions 54 and 72) at marker Cntg10192 that are the most significantly associated with dieback resistance are synonymous, coding the same amino acid. Assuming that marker Cntg10192 is not directly involved in the resistance, it is probable that a recombinant genotype will eventually be identified. On the other hand, marker-trait associations can be very strong between some tightly linked alleles. For example, Rick and Forbes [51] documented linkage between allozyme *Aps*^1 ^and tomato resistance gene *Mi *that did not break in as many as 30 backcross generations.

Chromosomal linkage group 2 contains a large cluster of resistance genes that confer resistance to downy mildew (*Bremia lactucae*) (*Dm1*, *Dm3*, *Dm6*, *Dm14*, *Dm15*, *Dm16*, *Dm18*) and lettuce root aphid (*Ra*) [52,53]. However, the Cntg10192 marker is well separated (> 25 cM) from this cluster on the Salinas × UC96US12 map. Moreover, *Tvr1 *is one of the few resistance genes that was not at a genetic position coincident with any type of candidate resistance gene so far mapped in lettuce [52]. Thus, it is possible that *Tvr1 *is different from the common types of pathogen recognition genes.

### Using high-resolution DNA melting analysis for marker-assisted selection

We used HRM to directly detect sequence variations in PCR amplicons. High-resolution melting curves were recorded by the slow and steady heating of PCR products in a LightScanner instrument. Changes in the shape of the melting curve were then used to identify mutations and variations. The method worked well for most of the analyzed markers, however, in a few cases, alleles could not be distinguished. When this occurred, we applied two alternative approaches to increase sensitivity through heteroduplex formation. In one approach, the heteroduplex formation was facilitated through mixing of samples prior to PCR. For example, if one sample contained DNA from cv. Salinas only, the other one would contain a mix of DNA (1:1 ratio) from both cv. Salinas and Valmaine. The second alternative used an unlabeled probe 20 bp to 35 bp long that was designed for the region carrying the SNP. The probe was included in the PCR mix prior to cycling but was not consumed during amplification due to 3' block. Genotyping was accomplished by monitoring the melting of probe-target duplexes post-PCR as described in LightScanner manual. Both of the above alternatives improved allele detection; however, the probe-target duplex approach appeared to be more sensitive.

## Conclusion

Lettuce dieback is a soil-borne viral disease that is one of the limiting factors for romaine and leaf-type lettuce production in California. Currently, there is no method that effectively reduces, removes, or destroys the virus in infested soil. Thus the best control of lettuce dieback is accomplished by using resistant cultivars. However, development of resistant cultivars up to now has required extensive field-based testing. Our identification of a molecular marker that is tightly linked to the *Tvr1 *gene conferring durable resistance will reduce the need for field-based screening and accelerate development of resistant cultivars.

A combination of classical linkage mapping and association mapping allowed us to pinpoint the location of the resistance gene on chromosomal linkage group 2. Examination of the *Tvr1 *region revealed a relatively high level of nucleotide polymorphism (for a selfing species) and extensive linkage disequilibrium. One of the markers (Cntg10192) flanking the *Tvr1 *gene showed 100% accuracy in detecting resistant and susceptible phenotypes in a set of 200 *L. sativa *accessions from all horticultural types of lettuce and two accessions from *L. serriola*. A combination of three SNPs in this EST-based marker identified four haplotypes. Three of the haplotypes are related to dieback resistance, while a single haplotype is always associated with susceptibility to the disease.

Application of high-resolution DNA melting analysis allowed us to distinguish all four haplotypes of the Cntg10192 marker in a single assay. Since heterozygous state is also easily distinguishable by the HRM analysis (data not shown), we can identify and select homozygous individuals whose offspring do not segregate for resistance in the following generation. Screening for dieback resistance with this molecular marker is now part of our breeding program. Marker-assisted selection with Cntg10192 is being used to develop improved romaine and leaf-type cultivars resistant to the disease. In addition, we are employing the molecular markers to prevent inadvertent introgression of the susceptible haplotype into the iceberg lettuce gene pool.

### Data access

Described sequences have been submitted to GenBank under accession numbers GQ340976 to GQ341571.

## List of abbreviations

AFLP: amplified fragment length polymorphism; CGPDB: Compositae Genome Project Database; cntg (in marker name): contig; cv.: cultivar; EST: expressed sequence tag; HRM: high-resolution DNA melting curve analysis; indel: insertion or deletion; LD: linkage disequilibrium; NCBI: National Center for Biotechnology Information; PCR: polymerase chain reaction; RAPD: random amplification of polymorphic DNA; RIL: recombinant-inbred line; SFP: single-feature polymorphism; SNP: single-nucleotide polymorphism; VNTR: variable number tandem repeat.

## Authors' contributions

IS designed and coordinated the study, performed phenotypic evaluations, carried out statistical analyses of the data, and prepared the manuscript. DAP developed primers, performed marker and sequence analysis, and assisted in drafting the manuscript. LKM, MJT, OEO and RWM developed mapping populations, provided EST data, and revised the manuscript. BES carried out sequencing and revised the manuscript. All authors read and approved the final manuscript.
